# Synthesis, crystal structure and Hirshfeld surface analysis of 1-[(1-benzyl-1*H*-1,2,3-triazol-4-yl)meth­yl]-3-(2-oxo-2-phenyl­eth­yl)-1,3-di­hydro-2*H*-benzimidazol-2-one

**DOI:** 10.1107/S2056989026008492

**Published:** 2026-08-20

**Authors:** Asmaa Saber, Mohamed Srhir, Daouda Ballo, Tuncer Hökelek, Joel T. Mague, El Mokhtar Essassi, Nada Kheira Sebbar

**Affiliations:** a Higher Institute of Nursing Professions and Health Techniques Rabat, Morocco; b Laboratory of Heterocyclic Organic Chemistry Medicines Science Research Center Pharmacochemistry Competence Center Mohammed V University in Rabat Faculty of Sciences Av Ibn Battouta BP 1014 Rabat Morocco; c Laboratory of Constitution and Reaction of Matter (LCRM), UFR SSMT, Félix Houphouët Boigny University, 22 BP 582 Abidjan 22, Republic of Côte d’Ivoire; d Department of Physics, Hacettepe University, 06800 Beytepe, Ankara, Türkiye; e Department of Chemistry, Tulane University, New Orleans, LA 70118, USA; Vienna University of Technology, Austria

**Keywords:** crystal structure, triazole, benzimidazole, hydrogen bond, C—H⋯π(ring) inter­action

## Abstract

In the title mol­ecule, C_25_H_21_N_5_O_2_, the benzyl­triazole moiety and the phenyl portion of the 3-(2-oxo-2-phenyl­eth­yl) group are disordered over two sets of sites. In the crystal, layers of mol­ecules parallel to the *ab* plane are generated by C—H⋯O and C—H⋯N hydrogen bonds, enclosing *R*^2^_2_(10) and *R*^2^_2_(16) ring motifs, and C—H⋯π(ring) inter­actions. A Hirshfeld surface analysis of the crystal structure indicates that the most important contributions for the crystal packing are from H⋯H (41.3%), H⋯C/C⋯H (31.1%), H⋯O/O⋯H (13.2%) and H⋯N/N⋯H (10.7%) inter­actions.

## Abstract 2

In the title mol­ecule, the benzyl­triazole moiety and the phenyl portion of the 3-(2-oxo-2-phenyl­eth­yl) group are disordered over two sites. In the crystal, layers of mol­ecules parallel to the *ab* plane are generated by C—H⋯O and C—H⋯N hydrogen bonds, enclosing *R*^2^_2_(10) and *R*^2^_2_(16) ring motifs, and C—H⋯π(ring) inter­actions.

## 1. Chemical context

Benzimidazole is an important nitro­gen-containing heterocyclic compound. Owing to its unique structural and electronic properties, this bicyclic system is relevant in medicinal chemistry for the development of biologically active compounds. Numerous benzimidazole derivatives have demonstrated a broad spectrum of pharmacological activities, including anti­microbial, anti­viral, anti­parasitic, anti­cancer, anti-inflammatory, anti­oxidant, anti­histaminic, anti­ulcer and anti­diabetic effects, making this heterocycle an important structural motif in many therapeutic agents (Ansari & Lal, 2009 ▸; Navarrete-Vazquez *et al.*, 2001 ▸; Luo *et al.*, 2011 ▸; Hranjec *et al.*, 2006 ▸, 2011 ▸; Ramla *et al.*, 2007 ▸; Saber *et al.*, 2018 ▸; Solominova *et al.*, 2004 ▸). Among these derivatives, benzimidazol-2-ones have received particular attention because of their diverse biological properties, notably their activity as progesterone receptor antagonists and their promising therapeutic potential (Wang *et al.*, 2011 ▸).

**Figure 1:**
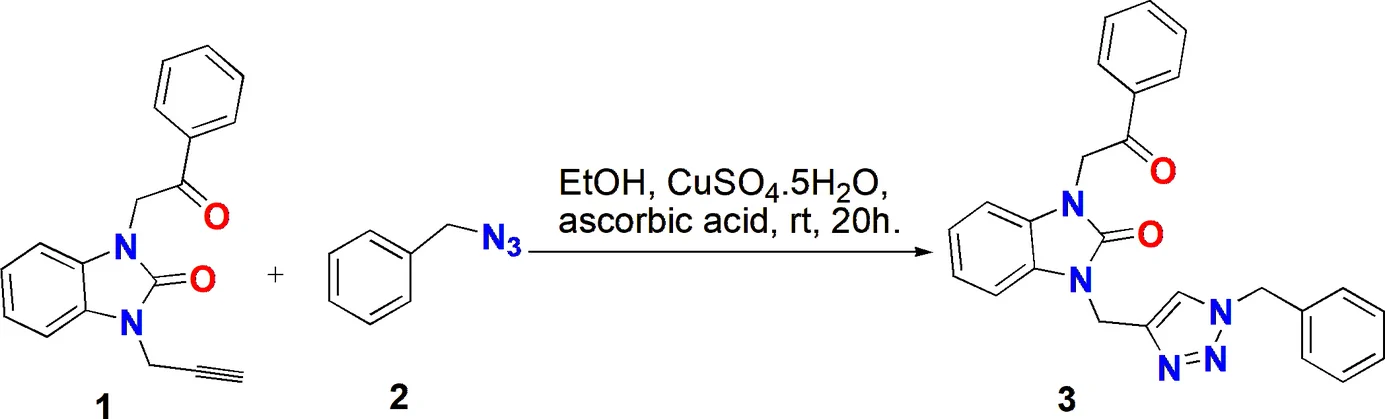
Synthesis scheme to obtain the title compound.

In a continuation of our studies on benzimidazole-based heterocycles incorporating a 1,2,3-triazole ring (Saber *et al.*, 2021 ▸; El Atrassi *et al.*, 2024 ▸), we synthesized a new triazole-functionalized benzimidazol-2-one derivative through the copper-catalyzed azide–alkyne cyclo­addition (CuAAC), a widely used click chemistry strategy. The reaction between 1-(prop-2-yn-1-yl)-3-(2-oxo-2-phenyl­eth­yl)-1,3-di­hydro-2*H*-benzimidazol-2-one and benzyl azide was carried out in a water/ethanol (1:1, *v*/*v*) mixture using copper(II) sulfate and sodium ascorbate as the catalytic system. Under these conditions, the title compound, C_25_H_21_N_5_O_2_, was obtained regioselectively in excellent yield (87%; Fig. 1 ▸). To gain further insight into its solid-state organization, the mol­ecular and crystal structures were determined by single-crystal X-ray diffraction, and the crystal packing was subsequently investigated by Hirshfeld surface analysis to identify the inter­molecular inter­actions governing the crystal stability.
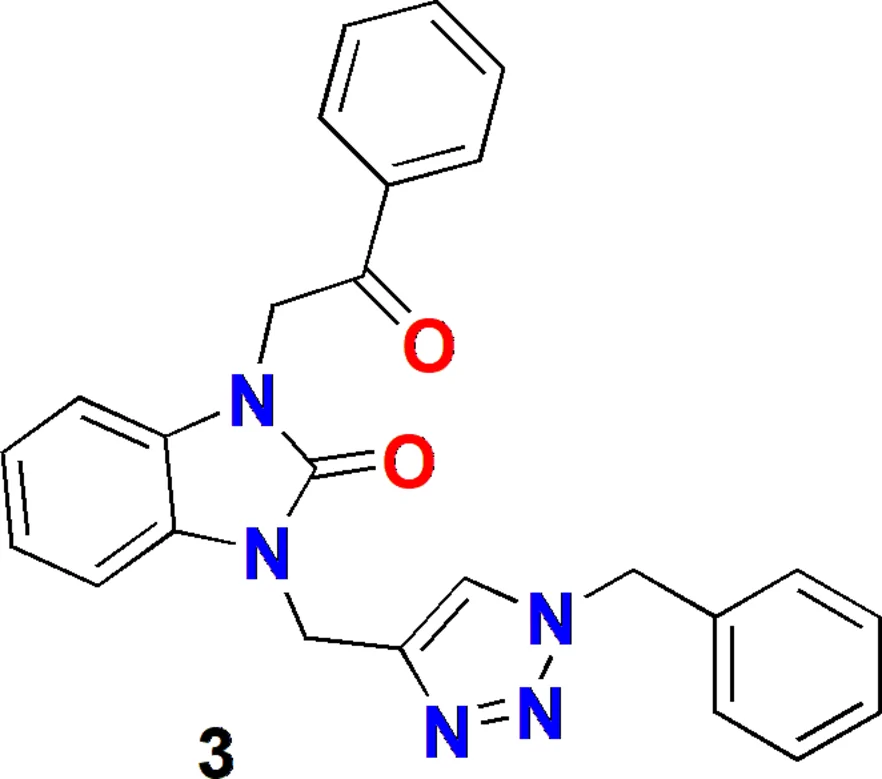


## 2. Structural commentary

**Figure 2:**
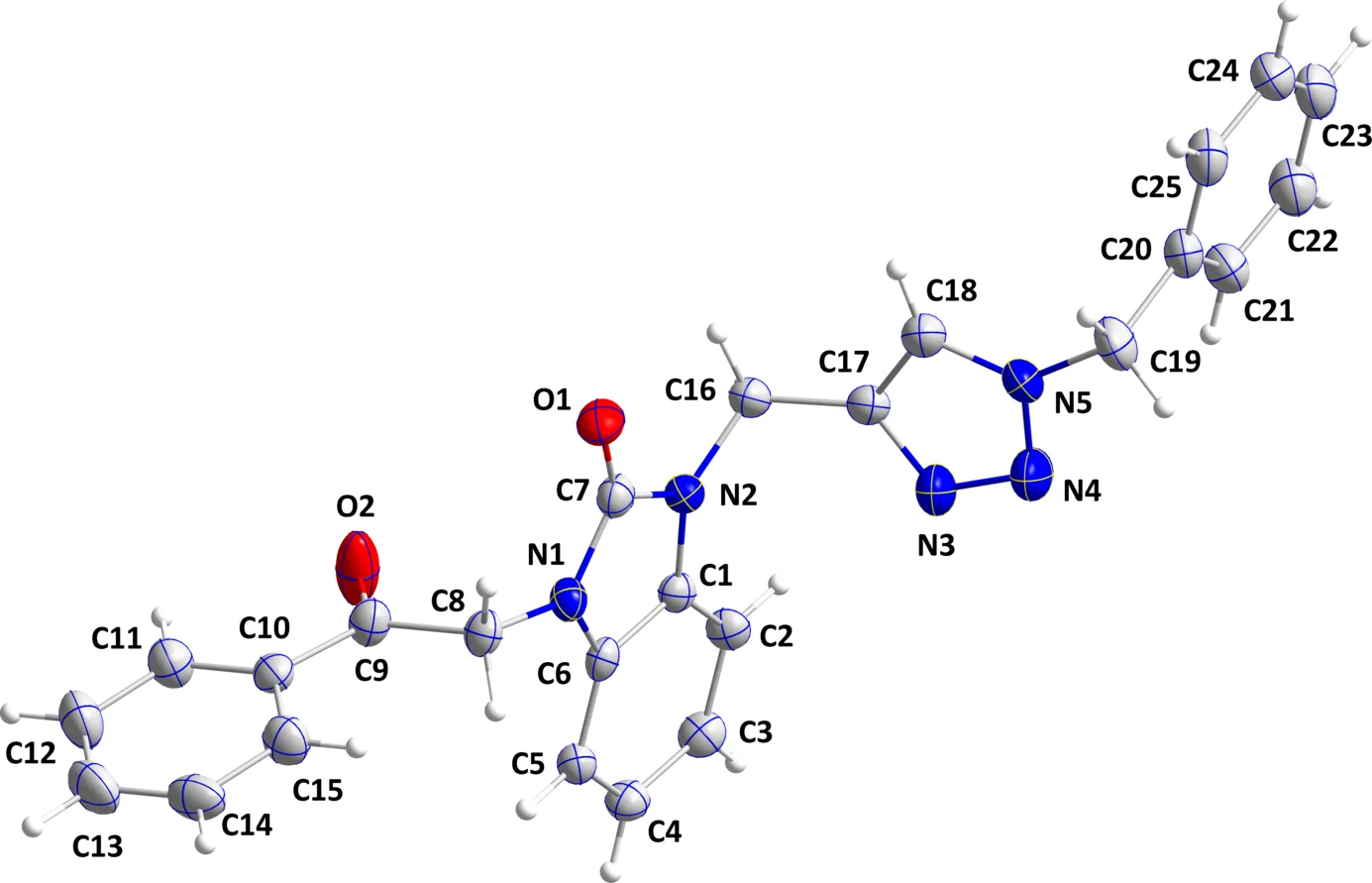
The title mol­ecule with displacement ellipsoids drawn at the 50% probability level. Only the major components of disorder are shown.

The mol­ecular structure is shown in Fig. 2 ▸. The benzimidazole unit is essentially planar with N1 and C7 being furthest from the least-squares plane at 0.0213 (14) and −0.0174 (15) Å, respectively (root-mean-square deviation = 0.0100 Å). The carbonyl group is slightly bent out of this plane as O1 is positioned −0.043 (2) Å from it. The benzyl­triazole substituent is disordered over two sets of nearly equally occupied sites, with the dihedral angle between the two orientations of the triazole ring being 64.7 (2)°. The dihedral angle between the mean planes of the benzimidazole unit and the C17/N3/N4/N5/C18 triazole ring is 68.69 (15)° while that between the latter ring plane and the mean plane of the C21–C25 ring is 75.05 (19)°. The corresponding angles for the minor component of this disorder are 77.36 (15) and 88.4 (2)°. The C10–C15 phenyl ring is also disordered over two sets of sites, with the dihedral angle between the mean planes of the benzimidazole unit and the major component of this disorder group being 83.07 (19)°. The dihedral angle between the two orientations of the C10–C15 phenyl ring is 18.4 (6)°.

## 3. Supra­molecular features

**Figure 3:**
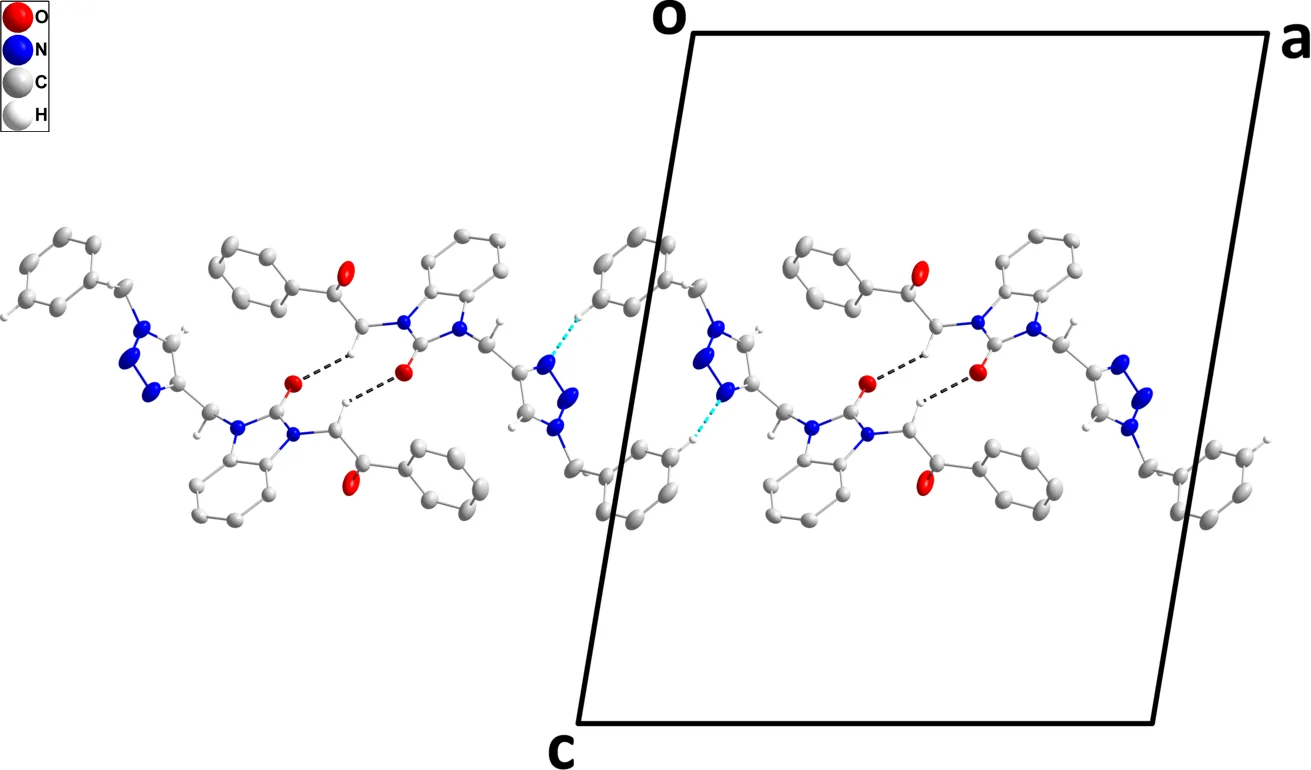
A portion of one hydrogen-bonded chain viewed along the *b-*axis direction with C—H⋯O and C—H⋯N hydrogen bonds depicted, respectively, by black and light-blue dashed lines. Only the major components of disorder are shown, and hydrogen atoms not involved in these inter­actions are omitted for clarity.

**Figure 4:**
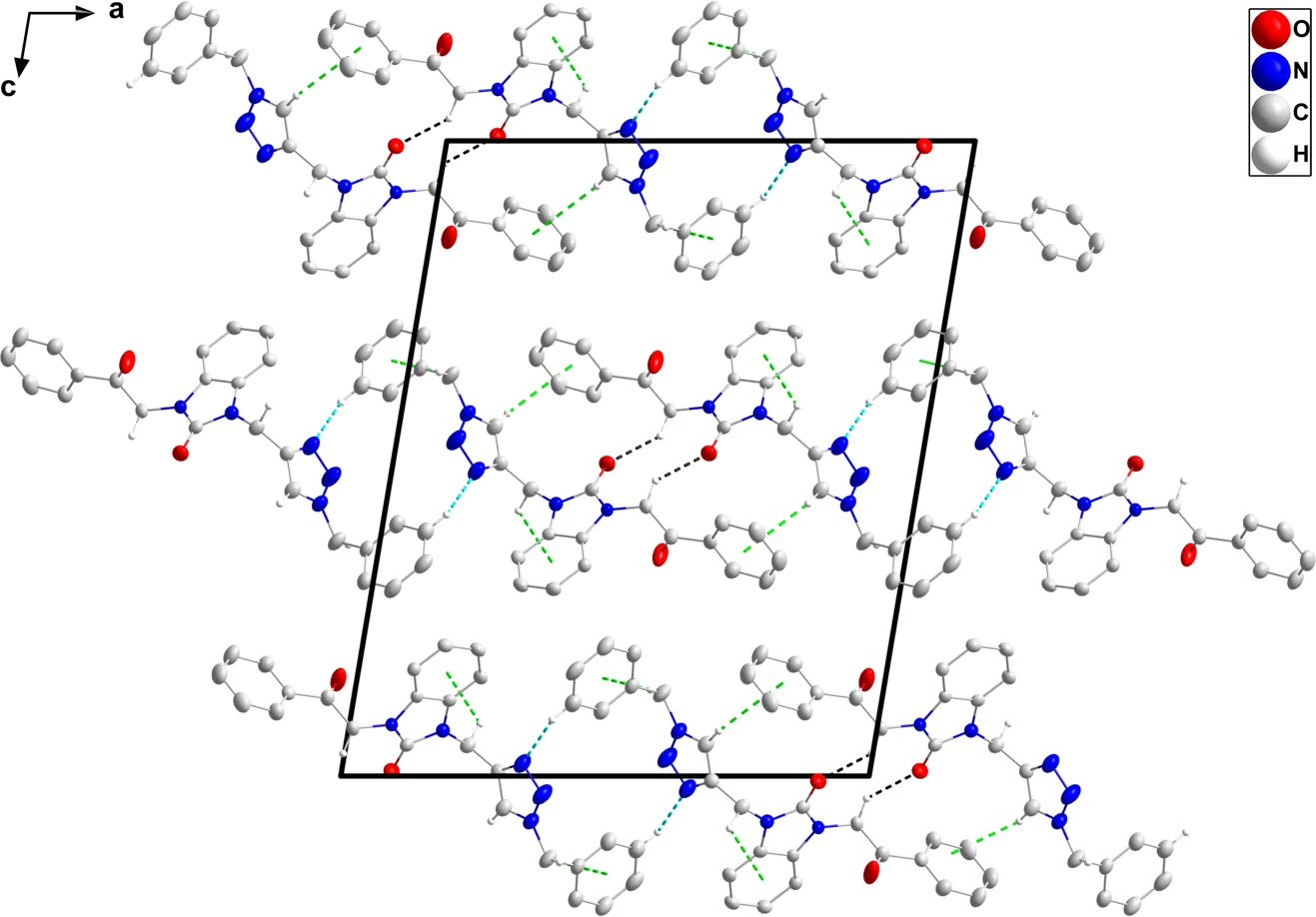
C—H⋯π(ring) inter­actions (green dashed lines) together with C—H⋯O and C—H⋯N hydrogen-bonding inter­actions form layers parallel to the *ab* plane.

In the crystal, C8—H8*A*⋯O1 and C22—H22⋯N3 hydrogen bonds (Table 1 ▸) form inversion dimers (Fig. 3 ▸), enclosing 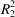
(10) and 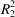
(16) ring motifs (Etter *et al.*, 1990 ▸), which are connected into chains extending parallel to the *a-*axis direction by inversion-related C18—H18⋯*Cg*5 inter­actions (Table 1 ▸, Fig. 4 ▸). The chains are linked along the *b-*axis direction by C16—H16B⋯*Cg*4 and C19—H19A⋯*Cg*6 inter­actions (Table 1 ▸), forming layers parallel to the *ab* plane (Figs. 4 ▸ and 5 ▸).

**Figure 6:**
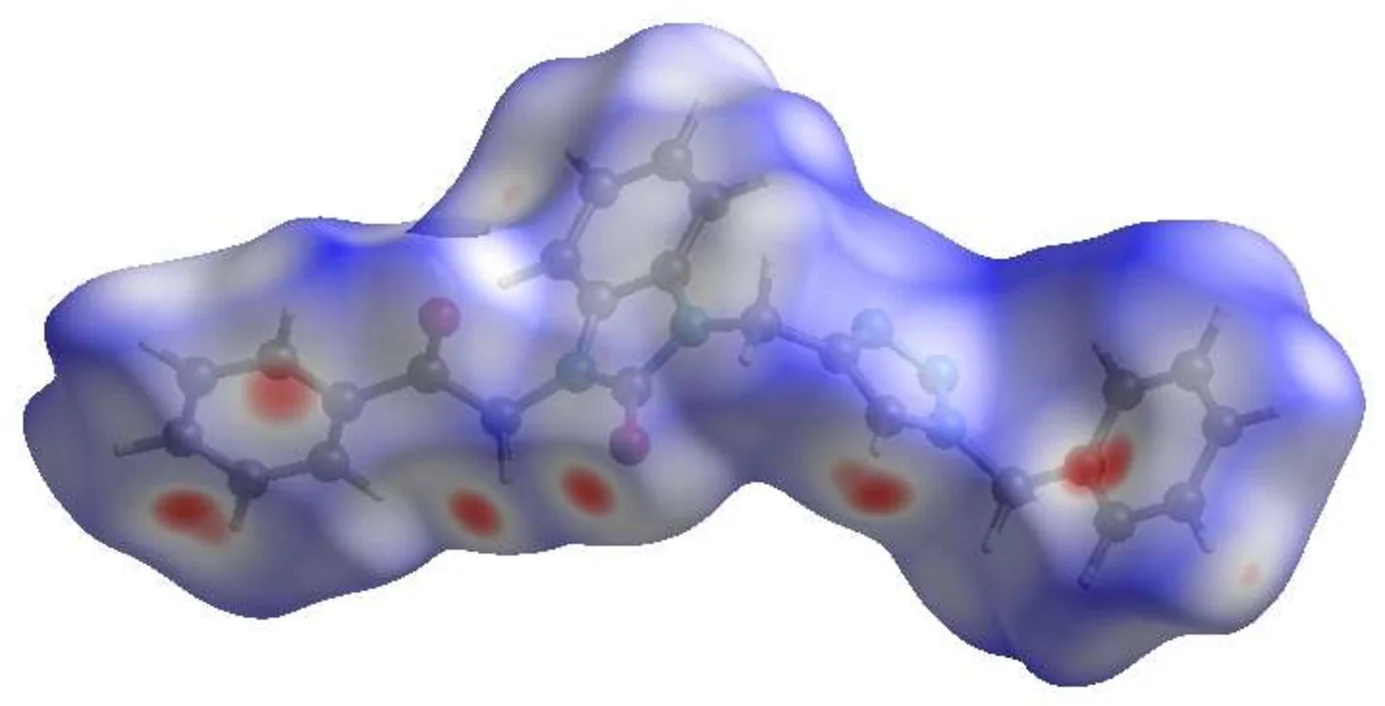
View of the three-dimensional Hirshfeld surface for mol­ecule plotted over *d*_norm_.

**Figure 7:**
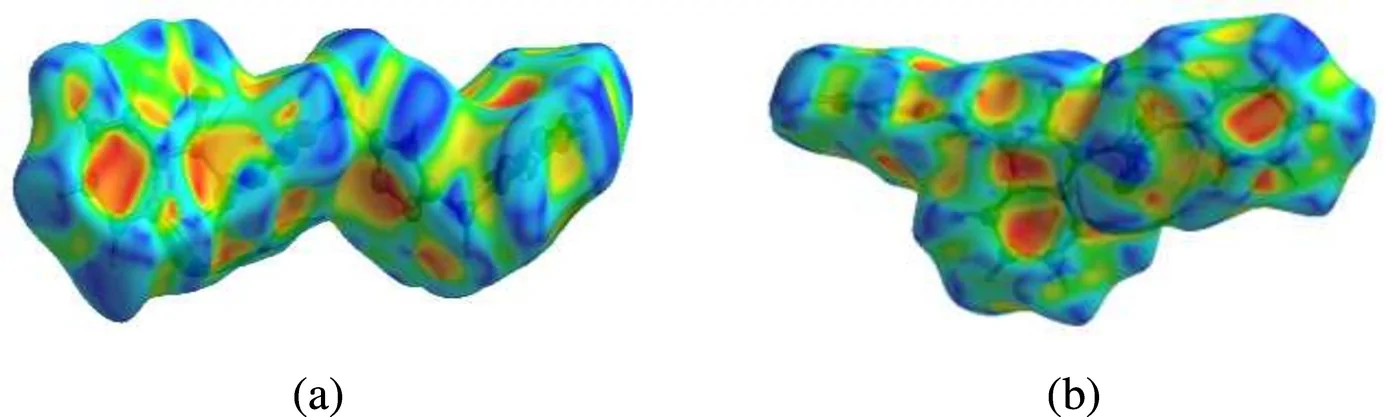
The shape-index surface showing two orientations for C—H⋯π(ring) inter­actions.

The inter­molecular inter­actions in the crystal were visualized by carrying out a Hirshfeld surface (HS) analysis using *CrystalExplorer* (Spackman *et al.*, 2021 ▸). It is noted that only the major components of the positionally disordered atoms were taken into account for the analysis. Fig. 6 ▸ shows the Hirshfeld surface with several neighboring mol­ecules in the crystal. The white surface indicates contacts with distances equal to the sum of van der Waals radii, and the red and blue colours indicate distances shorter (in close contact) or longer (distinct contacts) than the van der Waals radii, respectively. The red spots indicate their roles as the respective donors and/or acceptors atoms in hydrogen-bonding, as discussed above. The C—H⋯π(ring) inter­actions are shown in Fig. 7 ▸*a* and 7*b* by the presence of red π-holes.

**Figure 8:**
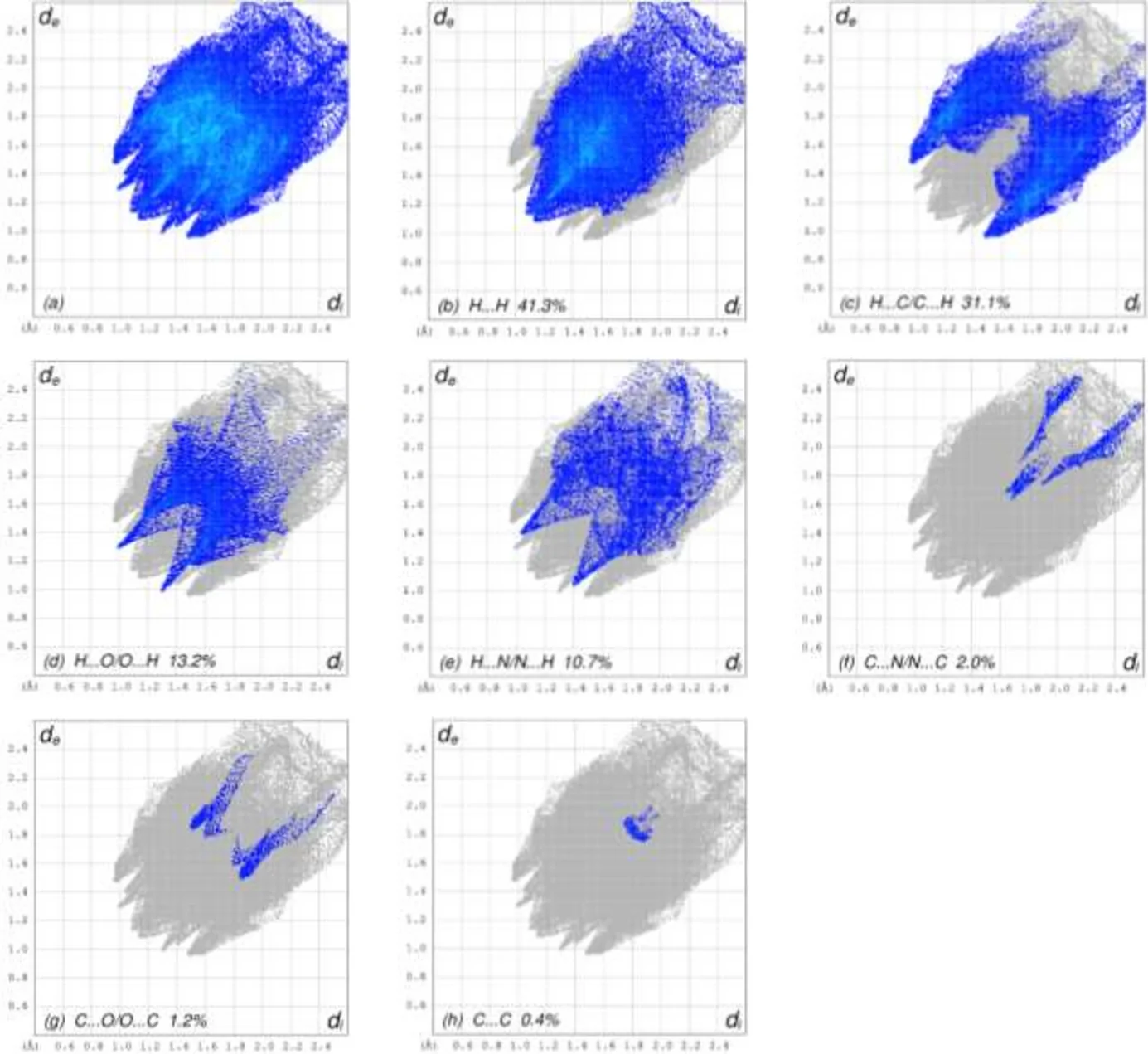
The two-dimensional fingerprint plots of the title compound (I), showing (*a*) all inter­actions, and delineated into (*b*) H⋯H, (*c*) H⋯C/C⋯H, (*d*) H⋯O/O⋯H, (*e*) H⋯N/N⋯H, (*f*) C⋯N/N⋯C, (*g*) C⋯O/O⋯C and (*h*) C⋯C inter­actions. The *d*_i_ and *d*_e_ values are the closest inter­nal and external distances (in Å) from given points on the Hirshfeld surface.

The overall two-dimensional fingerprint plot is shown in Fig. 8 ▸*a*, and those delineated into various contact types are illustrated in Fig. 8 ▸*b–h*. H⋯H, H⋯C/C⋯H, H⋯O/O⋯H and H⋯N/N⋯H contacts make the most significant contributions to the HS, at 41.3%, 31.1%, 13.2% and 10.7%, respectively.

## 4. Database survey

**Figure 9:**
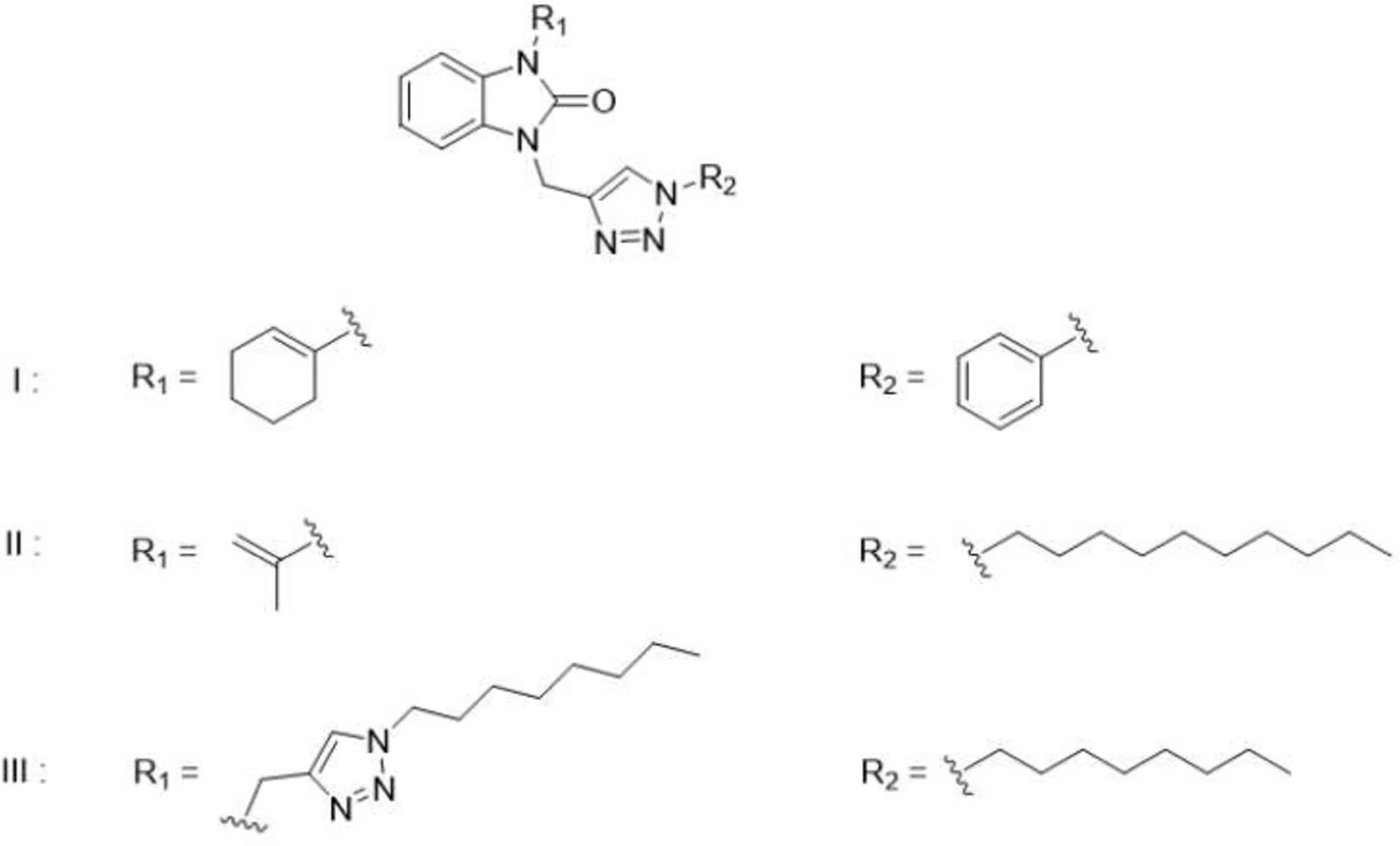
Schematic representation of closely related structures obtained from a database search.

A search of the Cambridge Structural Database (CSD, update July 2026; Groom *et al.*, 2016 ▸) revealed the presence of several structures closely related with the title compound, which are shown schematically in Fig. 9 ▸. These include **I** with *R*_1_ = *R*_2_ = –C_6_H_5_ (refcode PAZFOO; Adardour *et al.*, 2017 ▸), **II** with *R*_1_ = –C(CH_2_)=CH_2_, *R*_2_ = –(CH_2_)_9_CH_3_ (ETAJOB; Saber *et al.*, 2021 ▸), and **III** with *R*_1_ = –CH_2_—(C_2_HN_3_)—(CH_2_)_7_CH_3_, *R*_2_ = –(CH_2_)_7_CH_3_ (YIVWUZ; Zouhair *et al.*, 2023 ▸). A comparison of these structures highlights the versatility of the benzimidazol-2-one-1,2,3-triazole moiety, which tolerates a wide range of substituents at the N1 and N2 positions. In particular, variations in the alkyl or aryl substituents (*R*_1_, *R*_2_) significantly influence the crystal packing, inter­molecular inter­actions, and hydrogen-bonding motifs, without altering the general conformation of the heterocyclic core. Such structural adaptability makes this scaffold a valuable platform for further mol­ecular modification and pharmacological optimization.

## 5. Synthesis and crystallization

A 1 mmol solution of 1-(prop-2-yn-1-yl)-3-(2-oxo-2-phenyl­eth­yl)-1,3-di­hydro-2*H*-benzimidazol-2-one and 1.5 mmol of 1-(azido­meth­yl)benzene were dissolved in 15 ml of ethanol. This solution was added to 0.5 mmol of CuSO_4_ and 1 mmol of sodium ascorbate, dissolved in 15 ml of distilled water. The reaction mixture was stirred for 24 h at room temperature and then monitored by TLC until completion of the reaction. After filtration and concentration of the solution under reduced pressure, the residue was chromatographed on a silica gel column using an ethyl acetate/hexane (2:8 *v*/*v*) mixture as the eluent. The solid obtained on concentration of the eluate was filtered off, washed with water, dried, and then recrystallized from ethanol (87% yield).

## 6. Refinement

Crystal data, data collection and structure refinement details are summarized in Table 2 ▸. C-bound H atoms were positioned geometrically (C—H = 0.95–0.99 Å) and included as riding with isotropic displacement parameters 1.2–1.5 times those of the parent atoms. The benzyl triazole substituent is disordered over two resolved sets of sites in a 0.5049 (14)/0.4951 (14) ratio, and the C10–C15 phenyl ring in a 0.568 (7)/0.432 (7) ratio.

## Supplementary Material

Crystal structure: contains datablock(s) global, I. DOI: 10.1107/S2056989026008492/wm5807sup1.cif

Structure factors: contains datablock(s) I. DOI: 10.1107/S2056989026008492/wm5807Isup3.hkl

Supporting information file. DOI: 10.1107/S2056989026008492/wm5807Isup4.cdx

Supporting information file. DOI: 10.1107/S2056989026008492/wm5807Isup4.cml

CCDC reference: 2581766

Additional supporting information:  crystallographic information; 3D view; checkCIF report

## Figures and Tables

**Figure 5:**
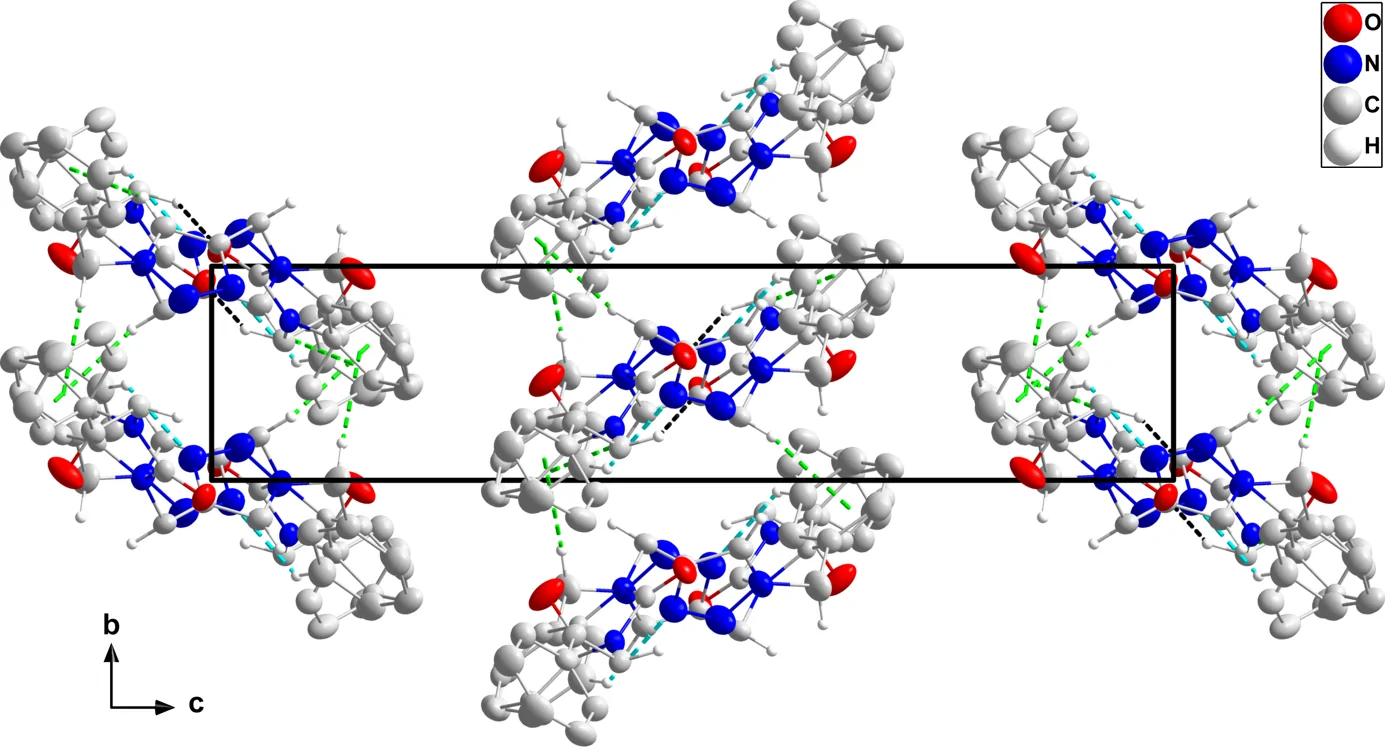
Packing plot in a view along the *a* axis showing the supra­molecular layers packed in an alternating mode along the *c* axis.

**Table 1:** Hydrogen-bond geometry (Å, °) *Cg*4, *Cg*5 and *Cg*6 are the centroids of the C1–C6, C10–C15 and C20–C25 benzene rings, respectively.

*D*—H⋯*A*	*D*—H	H⋯*A*	*D*⋯*A*	*D*—H⋯*A*
C8—H8*A*⋯O1^i^	0.99	2.37	3.190 (3)	140
C16—H16*B*⋯*Cg*4^ii^	0.99	2.69	3.384 (14)	128
C18—H18⋯*Cg*5^i^	0.95	2.88	3.700 (5)	146
C19—H19*A*⋯*Cg*6^iii^	0.99	2.71	3.678 (5)	166
C22—H22⋯N3^iv^	0.95	2.53	3.224 (5)	130

Symmetry codes: (i) 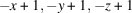; (ii) 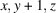; (iii) 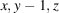; (iv) 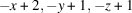.

**Table 2:** Experimental details

Crystal data	
Chemical formula	C_25_H_21_N_5_O_2_
*M*_r_	423.47
Crystal system, space group	Monoclinic, *P*2_1_/*n*
Temperature (K)	125
*a*, *b*, *c* (Å)	18.6129 (17), 4.9782 (5), 22.691 (2)
β (°)	99.479 (3)
*V* (Å^3^)	2073.8 (3)
*Z*	4
Radiation type	Mo *K*α
μ (mm^−1^)	0.09
Crystal size (mm)	0.36 × 0.21 × 0.10

Data collection	
Diffractometer	Bruker D8 QUEST PHOTON 3 diffractometer
Absorption correction	Multi-scan (*TWINABS*; Sheldrick, 2009 ▸)
*T*_min_, *T*_max_	0.97, 0.99
No. of measured, independent and observed [*I* > 2σ(*I*)] reflections	63938, 8059, 6008
*R*_int_	0.045
(sin θ/λ)_max_ (Å^−1^)	0.667

Refinement	
*R*[*F*^2^ > 2σ(*F*^2^)], *wR*(*F*^2^), *S*	0.053, 0.134, 1.03
No. of reflections	8059
No. of parameters	301
No. of restraints	24
H-atom treatment	H-atom parameters constrained
Δρ_max_, Δρ_min_ (e Å^−3^)	0.27, −0.29

Computer programs: *APEX4* and *SAINT* (Bruker, 2021 ▸), *SHELXT* (Sheldrick, 2015*a* ▸), *SHELXL* (Sheldrick, 2015*b* ▸), *DIAMOND* (Brandenburg & Putz, 2012 ▸) and *publCIF* (Westrip, 2010 ▸).

## supplementary crystallographic information

### 1-[(1-Benzyl-1H-1,2,3-triazol-4-yl)methyl]-3-(2-oxo-2-phenylethyl)-1,3-dihydro-2H-benzimidazol-2-one Crystal data

**Table:**

C_25_H_21_N_5_O_2_	*F*(000) = 888
*M**_r_* = 423.47	*D*_x_ = 1.356 Mg m^−^^3^
Monoclinic, *P*2_1_/*n*	Mo *K*α radiation, λ = 0.71073 Å
*a* = 18.6129 (17) Å	Cell parameters from 9948 reflections
*b* = 4.9782 (5) Å	θ = 2.5–28.2°
*c* = 22.691 (2) Å	µ = 0.09 mm^−^^1^
β = 99.479 (3)°	*T* = 125 K
*V* = 2073.8 (3) Å^3^	Column, colourless
*Z* = 4	0.36 × 0.21 × 0.10 mm

### 1-[(1-Benzyl-1H-1,2,3-triazol-4-yl)methyl]-3-(2-oxo-2-phenylethyl)-1,3-dihydro-2H-benzimidazol-2-one Data collection

**Table:**

Bruker D8 QUEST PHOTON 3 diffractometer	8059 independent reflections
Radiation source: fine-focus sealed tube	6008 reflections with *I* > 2σ(*I*)
Graphite monochromator	*R*_int_ = 0.045
Detector resolution: 7.3910 pixels mm^-1^	θ_max_ = 28.3°, θ_min_ = 2.2°
φ and ω scans	*h* = −24→24
Absorption correction: multi-scan (*TWINABS*; Sheldrick, 2009)	*k* = 0→6
*T*_min_ = 0.97, *T*_max_ = 0.99	*l* = 0→30
63938 measured reflections	

### 1-[(1-Benzyl-1H-1,2,3-triazol-4-yl)methyl]-3-(2-oxo-2-phenylethyl)-1,3-dihydro-2H-benzimidazol-2-one Refinement

**Table:**

Refinement on *F*^2^	Primary atom site location: dual
Least-squares matrix: full	Secondary atom site location: difference Fourier map
*R*[*F*^2^ > 2σ(*F*^2^)] = 0.053	Hydrogen site location: inferred from neighbouring sites
*wR*(*F*^2^) = 0.134	H-atom parameters constrained
*S* = 1.03	*w* = 1/[σ^2^(*F*_o_^2^) + (0.0462*P*)^2^ + 1.2404*P*] where *P* = (*F*_o_^2^ + 2*F*_c_^2^)/3
8059 reflections	(Δ/σ)_max_ < 0.001
301 parameters	Δρ_max_ = 0.27 e Å^−^^3^
24 restraints	Δρ_min_ = −0.29 e Å^−^^3^

### 1-[(1-Benzyl-1H-1,2,3-triazol-4-yl)methyl]-3-(2-oxo-2-phenylethyl)-1,3-dihydro-2H-benzimidazol-2-one Special details

**Table:**

Experimental. The diffraction data were obtained from 8 sets of frames, each of width 0.5° in ω or φ, collected with scan parameters determined by the "strategy" routine in *APEX4*. The scan time was 25 sec/frame. Analysis of 1004 reflections having I/σ(I) > 20 and chosen from the full data set with *CELL_NOW* (Sheldrick, 2008) showed the crystal to belong to the triclinic system and to be twinned by a 180° rotation about *a*. The raw data were processed using the multi-component version of *SAINT* under control of the two-component orientation file generated by *CELL_NOW*.
Geometry. All esds (except the esd in the dihedral angle between two l.s. planes) are estimated using the full covariance matrix. The cell esds are taken into account individually in the estimation of esds in distances, angles and torsion angles; correlations between esds in cell parameters are only used when they are defined by crystal symmetry. An approximate (isotropic) treatment of cell esds is used for estimating esds involving l.s. planes.
Refinement. Refinement of F^2^ against ALL reflections. The weighted R-factor wR and goodness of fit S are based on F^2^, conventional R-factors R are based on F, with F set to zero for negative F^2^. The threshold expression of F^2^ > 2sigma(F^2^) is used only for calculating R-factors(gt) etc. and is not relevant to the choice of reflections for refinement. R-factors based on F^2^ are statistically about twice as large as those based on F, and R- factors based on ALL data will be even larger. H-atoms attached to carbon were placed in calculated positions (C—H = 0.95 - 0.99 Å). All were included as riding contributions with isotropic displacement parameters 1.2 - 1.5 times those of the attached atoms. The substituent attached to N2 is disordered over two resolved sites in a 0.5049 (14)/0.4951 (14) ratio while the C10···C15 phenyl group is disordered over two closely spaced sites in a 0.568 (7)/0.432 (7) ratio. The disordered phenyl rings were refined as rigid hexagons while the remaider of the disordered atoms were refined with restraints making their geometries comparable. The model was refined as a 2-component twin. One reflection affected by the beamstop was omitted from the final refinement.

### 1-[(1-Benzyl-1H-1,2,3-triazol-4-yl)methyl]-3-(2-oxo-2-phenylethyl)-1,3-dihydro-2H-benzimidazol-2-one Fractional atomic coordinates and isotropic or equivalent isotropic displacement parameters (Å^2^)

**Table:**

	*x*	*y*	*z*	*U*_iso_*/*U*_eq_	Occ. (<1)
O1	0.59464 (8)	0.5795 (3)	0.49188 (7)	0.0374 (4)	
O2	0.46510 (9)	0.4659 (4)	0.34829 (9)	0.0562 (5)	
N1	0.57999 (8)	0.2455 (4)	0.41899 (7)	0.0263 (4)	
N2	0.67885 (9)	0.4979 (3)	0.42825 (7)	0.0266 (4)	
C1	0.68187 (10)	0.3221 (4)	0.38105 (8)	0.0237 (4)	
C2	0.73333 (11)	0.2867 (4)	0.34421 (9)	0.0274 (4)	
H2	0.776255	0.393023	0.348420	0.033*	
C3	0.71942 (11)	0.0889 (5)	0.30084 (9)	0.0308 (5)	
H3	0.753757	0.058981	0.274855	0.037*	
C4	0.65664 (11)	−0.0668 (5)	0.29429 (9)	0.0294 (5)	
H4	0.648729	−0.198764	0.263669	0.035*	
C5	0.60504 (10)	−0.0331 (4)	0.33180 (9)	0.0258 (4)	
H5	0.562432	−0.141034	0.327937	0.031*	
C6	0.61884 (10)	0.1642 (4)	0.37471 (8)	0.0233 (4)	
C7	0.61522 (11)	0.4566 (5)	0.45099 (9)	0.0274 (4)	
C8	0.50801 (10)	0.1541 (5)	0.42476 (9)	0.0281 (5)	
H8A	0.497440	0.204870	0.464639	0.034*	
H8B	0.506213	−0.044253	0.421756	0.034*	
C9	0.45034 (11)	0.2735 (5)	0.37696 (10)	0.0317 (5)	
C10	0.37482 (13)	0.1625 (9)	0.3687 (5)	0.0282 (6)	0.568 (7)
C11	0.3235 (3)	0.2743 (11)	0.3240 (4)	0.0384 (10)	0.568 (7)
H11	0.335088	0.430738	0.303564	0.046*	0.568 (7)
C12	0.2551 (2)	0.1571 (14)	0.3093 (3)	0.0496 (8)	0.568 (7)
H12	0.219955	0.233520	0.278776	0.060*	0.568 (7)
C13	0.23804 (17)	−0.0718 (12)	0.3393 (3)	0.0496 (16)	0.568 (7)
H13	0.191294	−0.151896	0.329190	0.059*	0.568 (7)
C14	0.2894 (3)	−0.1836 (10)	0.3839 (3)	0.0445 (12)	0.568 (7)
H14	0.277765	−0.340096	0.404392	0.053*	0.568 (7)
C15	0.3578 (2)	−0.0665 (10)	0.3986 (3)	0.0373 (10)	0.568 (7)
H15	0.392899	−0.142881	0.429181	0.045*	0.568 (7)
C10A	0.37745 (16)	0.1405 (13)	0.3662 (6)	0.0282 (6)	0.432 (7)
C11A	0.3257 (4)	0.2316 (17)	0.3192 (6)	0.0384 (10)	0.432 (7)
H11A	0.339678	0.347619	0.289896	0.046*	0.432 (7)
C12A	0.2534 (3)	0.153 (2)	0.3152 (4)	0.0496 (8)	0.432 (7)
H12A	0.217974	0.215097	0.283118	0.060*	0.432 (7)
C13A	0.23286 (17)	−0.0170 (18)	0.3582 (4)	0.0496 (16)	0.432 (7)
H13A	0.183453	−0.070791	0.355398	0.059*	0.432 (7)
C14A	0.2847 (4)	−0.1081 (15)	0.4051 (3)	0.0445 (12)	0.432 (7)
H14A	0.270635	−0.224158	0.434457	0.053*	0.432 (7)
C15A	0.3569 (3)	−0.0294 (14)	0.4091 (5)	0.0373 (10)	0.432 (7)
H15A	0.392339	−0.091638	0.441236	0.045*	0.432 (7)
C16	0.7310 (6)	0.7033 (9)	0.4515 (8)	0.0310 (7)	0.5049 (14)
H16A	0.706018	0.837559	0.473158	0.037*	0.5049 (14)
H16B	0.747108	0.796182	0.417352	0.037*	0.5049 (14)
C17	0.7965 (2)	0.6045 (8)	0.4922 (2)	0.0282 (5)	0.5049 (14)
N3	0.83961 (19)	0.3988 (8)	0.48012 (15)	0.0435 (8)	0.5049 (14)
N4	0.8876 (2)	0.3476 (8)	0.5290 (2)	0.0527 (13)	0.5049 (14)
N5	0.87433 (17)	0.5196 (7)	0.57097 (14)	0.0355 (6)	0.5049 (14)
C18	0.8187 (2)	0.6813 (8)	0.55005 (17)	0.0349 (7)	0.5049 (14)
H18	0.798766	0.820532	0.571027	0.042*	0.5049 (14)
C19	0.9184 (2)	0.5069 (10)	0.63041 (18)	0.0472 (9)	0.5049 (14)
H19A	0.941253	0.327202	0.635861	0.057*	0.5049 (14)
H19B	0.885808	0.526409	0.660527	0.057*	0.5049 (14)
C20	0.97723 (16)	0.7168 (7)	0.64224 (15)	0.0338 (11)	0.5049 (14)
C21	1.02722 (18)	0.7422 (7)	0.60332 (13)	0.0410 (10)	0.5049 (14)
H21	1.022982	0.632358	0.568691	0.049*	0.5049 (14)
C22	1.08340 (17)	0.9284 (8)	0.61508 (15)	0.0463 (10)	0.5049 (14)
H22	1.117562	0.945834	0.588479	0.056*	0.5049 (14)
C23	1.0896 (2)	1.0892 (9)	0.66575 (18)	0.0479 (15)	0.5049 (14)
H23	1.127994	1.216456	0.673782	0.057*	0.5049 (14)
C24	1.0396 (2)	1.0637 (9)	0.70467 (16)	0.0442 (14)	0.5049 (14)
H24	1.043848	1.173602	0.739298	0.053*	0.5049 (14)
C25	0.98343 (17)	0.8775 (8)	0.69291 (14)	0.0420 (10)	0.5049 (14)
H25	0.949267	0.860127	0.719511	0.050*	0.5049 (14)
C16A	0.7324 (6)	0.7019 (9)	0.4493 (8)	0.0310 (7)	0.4951 (14)
H16C	0.710760	0.836050	0.473364	0.037*	0.4951 (14)
H16D	0.747089	0.795638	0.414650	0.037*	0.4951 (14)
C17A	0.7975 (2)	0.5787 (9)	0.48617 (19)	0.0282 (5)	0.4951 (14)
N3A	0.80361 (19)	0.3171 (8)	0.50294 (16)	0.0435 (8)	0.4951 (14)
N4A	0.8704 (2)	0.2774 (9)	0.53272 (19)	0.0527 (13)	0.4951 (14)
N5A	0.90525 (17)	0.5125 (8)	0.53429 (15)	0.0355 (6)	0.4951 (14)
C18A	0.8623 (2)	0.7036 (8)	0.50613 (18)	0.0349 (7)	0.4951 (14)
H18A	0.874269	0.886763	0.501080	0.042*	0.4951 (14)
C19A	0.9810 (2)	0.5279 (11)	0.5637 (2)	0.0472 (9)	0.4951 (14)
H19C	1.011023	0.594346	0.534603	0.057*	0.4951 (14)
H19D	0.998082	0.344738	0.575901	0.057*	0.4951 (14)
C20A	0.99296 (19)	0.7077 (7)	0.61788 (13)	0.0338 (11)	0.4951 (14)
C21A	0.94870 (16)	0.6821 (7)	0.66129 (15)	0.0410 (10)	0.4951 (14)
H21A	0.909114	0.559270	0.655619	0.049*	0.4951 (14)
C22A	0.96238 (19)	0.8363 (9)	0.71299 (14)	0.0463 (10)	0.4951 (14)
H22A	0.932132	0.818855	0.742658	0.056*	0.4951 (14)
C23A	1.0203 (2)	1.0161 (9)	0.72129 (16)	0.0479 (15)	0.4951 (14)
H23A	1.029649	1.121515	0.756622	0.057*	0.4951 (14)
C24A	1.0646 (2)	1.0417 (9)	0.67788 (18)	0.0442 (14)	0.4951 (14)
H24A	1.104147	1.164592	0.683547	0.053*	0.4951 (14)
C25A	1.05088 (17)	0.8875 (8)	0.62617 (14)	0.0420 (10)	0.4951 (14)
H25A	1.081129	0.905010	0.596508	0.050*	0.4951 (14)

### 1-[(1-Benzyl-1H-1,2,3-triazol-4-yl)methyl]-3-(2-oxo-2-phenylethyl)-1,3-dihydro-2H-benzimidazol-2-one Atomic displacement parameters (Å^2^)

**Table:**

	*U*^11^	*U*^22^	*U*^33^	*U*^12^	*U*^13^	*U*^23^
O1	0.0361 (8)	0.0431 (10)	0.0337 (8)	0.0072 (8)	0.0074 (7)	−0.0104 (8)
O2	0.0307 (9)	0.0607 (13)	0.0737 (13)	−0.0057 (9)	−0.0014 (8)	0.0361 (11)
N1	0.0212 (8)	0.0335 (10)	0.0245 (8)	0.0018 (8)	0.0044 (6)	−0.0023 (8)
N2	0.0260 (8)	0.0261 (9)	0.0273 (8)	0.0014 (8)	0.0036 (7)	−0.0015 (7)
C1	0.0264 (10)	0.0226 (10)	0.0212 (9)	0.0018 (8)	0.0014 (7)	0.0028 (8)
C2	0.0284 (10)	0.0262 (11)	0.0286 (10)	−0.0029 (9)	0.0072 (8)	0.0027 (9)
C3	0.0334 (11)	0.0339 (12)	0.0270 (10)	0.0016 (10)	0.0107 (8)	0.0010 (10)
C4	0.0325 (11)	0.0301 (11)	0.0259 (10)	0.0023 (10)	0.0051 (8)	−0.0029 (9)
C5	0.0235 (9)	0.0266 (11)	0.0268 (10)	0.0000 (9)	0.0026 (8)	0.0000 (9)
C6	0.0211 (9)	0.0258 (10)	0.0229 (9)	0.0049 (8)	0.0031 (7)	0.0051 (8)
C7	0.0255 (10)	0.0305 (11)	0.0251 (10)	0.0054 (9)	0.0010 (8)	0.0008 (9)
C8	0.0227 (9)	0.0323 (12)	0.0301 (10)	0.0022 (9)	0.0064 (8)	0.0030 (10)
C9	0.0267 (10)	0.0333 (12)	0.0351 (11)	0.0017 (9)	0.0049 (9)	0.0049 (10)
C10	0.0256 (10)	0.0294 (13)	0.0302 (12)	0.0021 (10)	0.0062 (9)	−0.0050 (12)
C11	0.0337 (12)	0.042 (2)	0.0383 (18)	−0.0026 (14)	0.0013 (11)	0.000 (2)
C12	0.0315 (12)	0.0591 (18)	0.0536 (19)	−0.0047 (13)	−0.0066 (12)	−0.0023 (15)
C13	0.0318 (15)	0.057 (3)	0.059 (4)	−0.0149 (18)	0.0058 (18)	−0.014 (3)
C14	0.0415 (17)	0.038 (3)	0.057 (4)	−0.011 (2)	0.017 (2)	−0.009 (2)
C15	0.0321 (12)	0.0388 (19)	0.042 (3)	−0.0047 (12)	0.0075 (13)	0.0009 (17)
C10A	0.0256 (10)	0.0294 (13)	0.0302 (12)	0.0021 (10)	0.0062 (9)	−0.0050 (12)
C11A	0.0337 (12)	0.042 (2)	0.0383 (18)	−0.0026 (14)	0.0013 (11)	0.000 (2)
C12A	0.0315 (12)	0.0591 (18)	0.0536 (19)	−0.0047 (13)	−0.0066 (12)	−0.0023 (15)
C13A	0.0318 (15)	0.057 (3)	0.059 (4)	−0.0149 (18)	0.0058 (18)	−0.014 (3)
C14A	0.0415 (17)	0.038 (3)	0.057 (4)	−0.011 (2)	0.017 (2)	−0.009 (2)
C15A	0.0321 (12)	0.0388 (19)	0.042 (3)	−0.0047 (12)	0.0075 (13)	0.0009 (17)
C16	0.0321 (11)	0.0259 (11)	0.0332 (14)	−0.0001 (9)	−0.0005 (10)	−0.0036 (10)
C17	0.0290 (11)	0.0252 (12)	0.0302 (12)	−0.0008 (10)	0.0039 (10)	−0.0031 (10)
N3	0.0352 (18)	0.044 (2)	0.047 (2)	0.0072 (15)	−0.0079 (13)	−0.0031 (16)
N4	0.044 (2)	0.051 (3)	0.0549 (16)	0.014 (2)	−0.0151 (15)	−0.0090 (17)
N5	0.0311 (15)	0.0365 (15)	0.0357 (15)	−0.0001 (13)	−0.0043 (10)	−0.0069 (14)
C18	0.0340 (16)	0.0297 (16)	0.0390 (17)	0.0012 (14)	−0.0003 (13)	−0.0024 (15)
C19	0.0358 (18)	0.055 (2)	0.0441 (19)	0.0007 (17)	−0.0118 (15)	−0.0084 (19)
C20	0.025 (2)	0.0375 (17)	0.037 (3)	0.0010 (17)	−0.0018 (19)	−0.001 (2)
C21	0.032 (2)	0.043 (2)	0.046 (2)	−0.0036 (19)	0.0001 (17)	−0.0064 (19)
C22	0.036 (2)	0.055 (3)	0.046 (2)	0.0001 (19)	0.0008 (17)	−0.001 (2)
C23	0.038 (3)	0.047 (3)	0.051 (3)	0.003 (2)	−0.013 (2)	0.001 (2)
C24	0.036 (3)	0.041 (2)	0.050 (3)	0.003 (2)	−0.008 (2)	−0.010 (3)
C25	0.0287 (19)	0.052 (3)	0.043 (2)	0.0044 (19)	0.0006 (16)	0.002 (2)
C16A	0.0321 (11)	0.0259 (11)	0.0332 (14)	−0.0001 (9)	−0.0005 (10)	−0.0036 (10)
C17A	0.0290 (11)	0.0252 (12)	0.0302 (12)	−0.0008 (10)	0.0039 (10)	−0.0031 (10)
N3A	0.0352 (18)	0.044 (2)	0.047 (2)	0.0072 (15)	−0.0079 (13)	−0.0031 (16)
N4A	0.044 (2)	0.051 (3)	0.0549 (16)	0.014 (2)	−0.0151 (15)	−0.0090 (17)
N5A	0.0311 (15)	0.0365 (15)	0.0357 (15)	−0.0001 (13)	−0.0043 (10)	−0.0069 (14)
C18A	0.0340 (16)	0.0297 (16)	0.0390 (17)	0.0012 (14)	−0.0003 (13)	−0.0024 (15)
C19A	0.0358 (18)	0.055 (2)	0.0441 (19)	0.0007 (17)	−0.0118 (15)	−0.0084 (19)
C20A	0.025 (2)	0.0375 (17)	0.037 (3)	0.0010 (17)	−0.0018 (19)	−0.001 (2)
C21A	0.032 (2)	0.043 (2)	0.046 (2)	−0.0036 (19)	0.0001 (17)	−0.0064 (19)
C22A	0.036 (2)	0.055 (3)	0.046 (2)	0.0001 (19)	0.0008 (17)	−0.001 (2)
C23A	0.038 (3)	0.047 (3)	0.051 (3)	0.003 (2)	−0.013 (2)	0.001 (2)
C24A	0.036 (3)	0.041 (2)	0.050 (3)	0.003 (2)	−0.008 (2)	−0.010 (3)
C25A	0.0287 (19)	0.052 (3)	0.043 (2)	0.0044 (19)	0.0006 (16)	0.002 (2)

### 1-[(1-Benzyl-1H-1,2,3-triazol-4-yl)methyl]-3-(2-oxo-2-phenylethyl)-1,3-dihydro-2H-benzimidazol-2-one Geometric parameters (Å, º)

**Table:**

O1—C7	1.224 (2)	C16—H16A	0.9900
O2—C9	1.214 (3)	C16—H16B	0.9900
N1—C7	1.380 (3)	C17—N3	1.357 (4)
N1—C6	1.391 (2)	C17—C18	1.363 (5)
N1—C8	1.441 (2)	N3—N4	1.329 (4)
N2—C7	1.383 (3)	N4—N5	1.334 (5)
N2—C1	1.391 (2)	N5—C18	1.336 (4)
N2—C16	1.448 (3)	N5—C19	1.460 (4)
N2—C16A	1.448 (3)	C18—H18	0.9500
C1—C2	1.382 (3)	C19—C20	1.506 (4)
C1—C6	1.400 (3)	C19—H19A	0.9900
C2—C3	1.386 (3)	C19—H19B	0.9900
C2—H2	0.9500	C20—C21	1.3900
C3—C4	1.390 (3)	C20—C25	1.3900
C3—H3	0.9500	C21—C22	1.3900
C4—C5	1.394 (3)	C21—H21	0.9500
C4—H4	0.9500	C22—C23	1.3900
C5—C6	1.377 (3)	C22—H22	0.9500
C5—H5	0.9500	C23—C24	1.3900
C8—C9	1.516 (3)	C23—H23	0.9500
C8—H8A	0.9900	C24—C25	1.3900
C8—H8B	0.9900	C24—H24	0.9500
C9—C10A	1.493 (2)	C25—H25	0.9500
C9—C10	1.493 (2)	C16A—C17A	1.488 (3)
C10—C11	1.3900	C16A—H16C	0.9900
C10—C15	1.3900	C16A—H16D	0.9900
C11—C12	1.3900	C17A—N3A	1.356 (5)
C11—H11	0.9500	C17A—C18A	1.366 (5)
C12—C13	1.3900	N3A—N4A	1.328 (4)
C12—H12	0.9500	N4A—N5A	1.336 (5)
C13—C14	1.3900	N5A—C18A	1.335 (4)
C13—H13	0.9500	N5A—C19A	1.459 (4)
C14—C15	1.3900	C18A—H18A	0.9500
C14—H14	0.9500	C19A—C20A	1.507 (4)
C15—H15	0.9500	C19A—H19C	0.9900
C10A—C11A	1.3900	C19A—H19D	0.9900
C10A—C15A	1.3900	C20A—C21A	1.3900
C11A—C12A	1.3900	C20A—C25A	1.3900
C11A—H11A	0.9500	C21A—C22A	1.3900
C12A—C13A	1.3900	C21A—H21A	0.9500
C12A—H12A	0.9500	C22A—C23A	1.3900
C13A—C14A	1.3900	C22A—H22A	0.9500
C13A—H13A	0.9500	C23A—C24A	1.3900
C14A—C15A	1.3900	C23A—H23A	0.9500
C14A—H14A	0.9500	C24A—C25A	1.3900
C15A—H15A	0.9500	C24A—H24A	0.9500
C16—C17	1.488 (3)	C25A—H25A	0.9500
			
C7—N1—C6	110.06 (16)	N2—C16—H16B	108.5
C7—N1—C8	124.12 (17)	C17—C16—H16B	108.5
C6—N1—C8	125.12 (17)	H16A—C16—H16B	107.5
C7—N2—C1	109.86 (17)	N3—C17—C18	108.1 (3)
C7—N2—C16	122.2 (9)	N3—C17—C16	125.1 (7)
C1—N2—C16	127.9 (9)	C18—C17—C16	126.4 (7)
C7—N2—C16A	124.5 (9)	N4—N3—C17	108.3 (3)
C1—N2—C16A	125.6 (9)	N3—N4—N5	107.1 (3)
C2—C1—N2	131.72 (19)	N4—N5—C18	110.9 (3)
C2—C1—C6	121.25 (18)	N4—N5—C19	119.3 (3)
N2—C1—C6	107.02 (17)	C18—N5—C19	129.7 (4)
C1—C2—C3	116.93 (19)	N5—C18—C17	105.5 (3)
C1—C2—H2	121.5	N5—C18—H18	127.2
C3—C2—H2	121.5	C17—C18—H18	127.2
C2—C3—C4	121.85 (19)	N5—C19—C20	114.6 (4)
C2—C3—H3	119.1	N5—C19—H19A	108.6
C4—C3—H3	119.1	C20—C19—H19A	108.6
C3—C4—C5	121.3 (2)	N5—C19—H19B	108.6
C3—C4—H4	119.4	C20—C19—H19B	108.6
C5—C4—H4	119.4	H19A—C19—H19B	107.6
C6—C5—C4	116.76 (19)	C21—C20—C25	120.0
C6—C5—H5	121.6	C21—C20—C19	119.6 (3)
C4—C5—H5	121.6	C25—C20—C19	120.4 (3)
C5—C6—N1	131.26 (18)	C22—C21—C20	120.0
C5—C6—C1	121.92 (18)	C22—C21—H21	120.0
N1—C6—C1	106.80 (17)	C20—C21—H21	120.0
O1—C7—N1	126.75 (19)	C23—C22—C21	120.0
O1—C7—N2	127.1 (2)	C23—C22—H22	120.0
N1—C7—N2	106.17 (17)	C21—C22—H22	120.0
N1—C8—C9	111.74 (17)	C22—C23—C24	120.0
N1—C8—H8A	109.3	C22—C23—H23	120.0
C9—C8—H8A	109.3	C24—C23—H23	120.0
N1—C8—H8B	109.3	C25—C24—C23	120.0
C9—C8—H8B	109.3	C25—C24—H24	120.0
H8A—C8—H8B	107.9	C23—C24—H24	120.0
O2—C9—C10A	122.8 (5)	C24—C25—C20	120.0
O2—C9—C10	121.1 (4)	C24—C25—H25	120.0
O2—C9—C8	119.78 (19)	C20—C25—H25	120.0
C10A—C9—C8	117.4 (4)	N2—C16A—C17A	110.4 (3)
C10—C9—C8	119.0 (4)	N2—C16A—H16C	109.6
C11—C10—C15	120.0	C17A—C16A—H16C	109.6
C11—C10—C9	117.6 (5)	N2—C16A—H16D	109.6
C15—C10—C9	121.9 (5)	C17A—C16A—H16D	109.6
C10—C11—C12	120.0	H16C—C16A—H16D	108.1
C10—C11—H11	120.0	N3A—C17A—C18A	108.4 (3)
C12—C11—H11	120.0	N3A—C17A—C16A	125.2 (4)
C13—C12—C11	120.0	C18A—C17A—C16A	126.3 (4)
C13—C12—H12	120.0	N4A—N3A—C17A	108.3 (3)
C11—C12—H12	120.0	N3A—N4A—N5A	107.0 (3)
C12—C13—C14	120.0	C18A—N5A—N4A	111.2 (3)
C12—C13—H13	120.0	C18A—N5A—C19A	129.7 (4)
C14—C13—H13	120.0	N4A—N5A—C19A	119.1 (3)
C13—C14—C15	120.0	N5A—C18A—C17A	105.1 (3)
C13—C14—H14	120.0	N5A—C18A—H18A	127.5
C15—C14—H14	120.0	C17A—C18A—H18A	127.5
C14—C15—C10	120.0	N5A—C19A—C20A	114.0 (3)
C14—C15—H15	120.0	N5A—C19A—H19C	108.8
C10—C15—H15	120.0	C20A—C19A—H19C	108.8
C11A—C10A—C15A	120.0	N5A—C19A—H19D	108.8
C11A—C10A—C9	118.5 (7)	C20A—C19A—H19D	108.8
C15A—C10A—C9	120.1 (8)	H19C—C19A—H19D	107.7
C10A—C11A—C12A	120.0	C21A—C20A—C25A	120.0
C10A—C11A—H11A	120.0	C21A—C20A—C19A	119.9 (3)
C12A—C11A—H11A	120.0	C25A—C20A—C19A	120.0 (3)
C13A—C12A—C11A	120.0	C22A—C21A—C20A	120.0
C13A—C12A—H12A	120.0	C22A—C21A—H21A	120.0
C11A—C12A—H12A	120.0	C20A—C21A—H21A	120.0
C12A—C13A—C14A	120.0	C21A—C22A—C23A	120.0
C12A—C13A—H13A	120.0	C21A—C22A—H22A	120.0
C14A—C13A—H13A	120.0	C23A—C22A—H22A	120.0
C15A—C14A—C13A	120.0	C24A—C23A—C22A	120.0
C15A—C14A—H14A	120.0	C24A—C23A—H23A	120.0
C13A—C14A—H14A	120.0	C22A—C23A—H23A	120.0
C14A—C15A—C10A	120.0	C23A—C24A—C25A	120.0
C14A—C15A—H15A	120.0	C23A—C24A—H24A	120.0
C10A—C15A—H15A	120.0	C25A—C24A—H24A	120.0
N2—C16—C17	115.1 (3)	C24A—C25A—C20A	120.0
N2—C16—H16A	108.5	C24A—C25A—H25A	120.0
C17—C16—H16A	108.5	C20A—C25A—H25A	120.0
			
C7—N2—C1—C2	179.6 (2)	C11A—C12A—C13A—C14A	0.0
C16—N2—C1—C2	1.3 (4)	C12A—C13A—C14A—C15A	0.0
C16A—N2—C1—C2	1.9 (4)	C13A—C14A—C15A—C10A	0.0
C7—N2—C1—C6	−0.8 (2)	C11A—C10A—C15A—C14A	0.0
C16—N2—C1—C6	−179.1 (3)	C9—C10A—C15A—C14A	166.5 (7)
C16A—N2—C1—C6	−178.4 (3)	C7—N2—C16—C17	101.9 (12)
N2—C1—C2—C3	179.8 (2)	C1—N2—C16—C17	−80.1 (15)
C6—C1—C2—C3	0.2 (3)	N2—C16—C17—N3	50.9 (18)
C1—C2—C3—C4	0.2 (3)	N2—C16—C17—C18	−121.0 (10)
C2—C3—C4—C5	−0.9 (3)	C18—C17—N3—N4	0.05 (10)
C3—C4—C5—C6	1.1 (3)	C16—C17—N3—N4	−173.1 (9)
C4—C5—C6—N1	−178.8 (2)	C17—N3—N4—N5	0.02 (10)
C4—C5—C6—C1	−0.7 (3)	N3—N4—N5—C18	−0.08 (19)
C7—N1—C6—C5	−179.1 (2)	N3—N4—N5—C19	178.9 (4)
C8—N1—C6—C5	−8.4 (3)	N4—N5—C18—C17	0.1 (2)
C7—N1—C6—C1	2.6 (2)	C19—N5—C18—C17	−178.8 (4)
C8—N1—C6—C1	173.32 (18)	N3—C17—C18—N5	−0.09 (18)
C2—C1—C6—C5	0.1 (3)	C16—C17—C18—N5	172.9 (8)
N2—C1—C6—C5	−179.59 (17)	N4—N5—C19—C20	101.2 (4)
C2—C1—C6—N1	178.60 (18)	C18—N5—C19—C20	−80.0 (5)
N2—C1—C6—N1	−1.1 (2)	N5—C19—C20—C21	−54.1 (5)
C6—N1—C7—O1	177.9 (2)	N5—C19—C20—C25	128.3 (4)
C8—N1—C7—O1	7.1 (3)	C25—C20—C21—C22	0.0
C6—N1—C7—N2	−3.1 (2)	C19—C20—C21—C22	−177.6 (4)
C8—N1—C7—N2	−173.90 (17)	C20—C21—C22—C23	0.0
C1—N2—C7—O1	−178.6 (2)	C21—C22—C23—C24	0.0
C16—N2—C7—O1	−0.3 (4)	C22—C23—C24—C25	0.0
C16A—N2—C7—O1	−1.0 (5)	C23—C24—C25—C20	0.0
C1—N2—C7—N1	2.4 (2)	C21—C20—C25—C24	0.0
C16—N2—C7—N1	−179.2 (3)	C19—C20—C25—C24	177.6 (4)
C16A—N2—C7—N1	−180.0 (4)	C7—N2—C16A—C17A	105.0 (12)
C7—N1—C8—C9	96.7 (2)	C1—N2—C16A—C17A	−77.8 (15)
C6—N1—C8—C9	−72.8 (3)	N2—C16A—C17A—N3A	−6 (2)
N1—C8—C9—O2	−15.4 (3)	N2—C16A—C17A—C18A	170.2 (7)
N1—C8—C9—C10A	162.8 (5)	C18A—C17A—N3A—N4A	0.02 (10)
N1—C8—C9—C10	168.5 (4)	C16A—C17A—N3A—N4A	176.8 (12)
O2—C9—C10—C11	4.7 (5)	C17A—N3A—N4A—N5A	−0.03 (10)
C8—C9—C10—C11	−179.3 (3)	N3A—N4A—N5A—C18A	0.02 (19)
O2—C9—C10—C15	176.3 (4)	N3A—N4A—N5A—C19A	−179.0 (4)
C8—C9—C10—C15	−7.6 (7)	N4A—N5A—C18A—C17A	0.0 (2)
C15—C10—C11—C12	0.0	C19A—N5A—C18A—C17A	178.9 (4)
C9—C10—C11—C12	171.8 (6)	N3A—C17A—C18A—N5A	−0.01 (18)
C10—C11—C12—C13	0.0	C16A—C17A—C18A—N5A	−176.7 (11)
C11—C12—C13—C14	0.0	C18A—N5A—C19A—C20A	65.1 (6)
C12—C13—C14—C15	0.0	N4A—N5A—C19A—C20A	−116.0 (4)
C13—C14—C15—C10	0.0	N5A—C19A—C20A—C21A	48.6 (5)
C11—C10—C15—C14	0.0	N5A—C19A—C20A—C25A	−135.3 (4)
C9—C10—C15—C14	−171.5 (7)	C25A—C20A—C21A—C22A	0.0
O2—C9—C10A—C11A	3.6 (7)	C19A—C20A—C21A—C22A	176.1 (4)
C8—C9—C10A—C11A	−174.6 (4)	C20A—C21A—C22A—C23A	0.0
O2—C9—C10A—C15A	−163.1 (4)	C21A—C22A—C23A—C24A	0.0
C8—C9—C10A—C15A	18.7 (7)	C22A—C23A—C24A—C25A	0.0
C15A—C10A—C11A—C12A	0.0	C23A—C24A—C25A—C20A	0.0
C9—C10A—C11A—C12A	−166.8 (8)	C21A—C20A—C25A—C24A	0.0
C10A—C11A—C12A—C13A	0.0	C19A—C20A—C25A—C24A	−176.1 (4)

### 1-[(1-Benzyl-1H-1,2,3-triazol-4-yl)methyl]-3-(2-oxo-2-phenylethyl)-1,3-dihydro-2H-benzimidazol-2-one Hydrogen-bond geometry (Å, º)

*Cg*4, *Cg*5 and *Cg*6 are the centroids of the C1–C6, C10–C15 and C20–C25 benzene rings, respectively.

**Table:**

*D*—H···*A*	*D*—H	H···*A*	*D*···*A*	*D*—H···*A*
C8—H8*A*···O1^i^	0.99	2.37	3.190 (3)	140
C16—H16*B*···*Cg*4^ii^	0.99	2.69	3.384 (14)	128
C18—H18···*Cg*5^i^	0.95	2.88	3.700 (5)	146
C19—H19*A*···*Cg*6^iii^	0.99	2.71	3.678 (5)	166
C22—H22···N3^iv^	0.95	2.53	3.224 (5)	130

Symmetry codes: (i) −*x*+1, −*y*+1, −*z*+1; (ii) *x*, *y*+1, *z*; (iii) *x*, *y*−1, *z*; (iv) −*x*+2, −*y*+1, −*z*+1.
